# The characteristics of high-quality health and social care for people on probation: Professional and lived experience perspectives

**DOI:** 10.1177/02645505231222961

**Published:** 2024-01-23

**Authors:** Coral Sirdifield, Thomas Parkhouse, Philip Mullen

**Affiliations:** University of Lincoln, Lincoln, UK; Revolving Doors, London, UK

**Keywords:** Probation, health, health inequalities, social care, quality improvement, criminal justice

## Abstract

We investigated the characteristics of high-quality health and social care for people on probation and how they might be measured. Online open-ended survey responses and focus group data were analysed using thematic analysis. Providing high-quality care involves an understanding of the population’s needs; tailoring practice to accommodate and meet individuals’ needs; inter-agency collaboration; and seeking and acting on lived experience input. High-quality care is evidence-based and accessible and produces positive outcomes for people on probation. Foundations for achieving these outcomes are supportive, trusting and consistent supervisory relationships (working alliances) with professional practice that is sufficiently resourced, compassionate and person-centred.

## Background

Contact with the probation service provides an opportunity for criminal justice and health and social care agencies to work together to support health improvements in people that are in poorer health when compared to the general population, socially excluded and that often only access care at crisis point (Brooker et al., 2009, 2012; Revolving Doors Agency, 2017). Health issues such as substance misuse can be drivers of crime. For example, a recent report stated that ‘half of all acquisitive crime may be drug related and it’s estimated that this costs the public and the public purse over £9 billion a year’ (HMIP and CQC, 2021: 5). Therefore, addressing unmet needs can contribute to reducing crime and re-offending. Poor health may also act as a barrier to engaging in other activities believed to support desistance, such as gaining employment (Revolving Doors Agency, 2017). Improving the health of people on probation therefore provides individual and societal-level benefits such as improved health outcomes, and reductions in avoidable use of emergency care, re-offending and health inequalities (Criminal Justice Joint Inspections England and Wales, 2021; Revolving Doors Agency, 2017). Probation practitioners’ role in relation to this in England and Wales is detailed in the National Probation Service (NPS) health and social care strategy^1^ (HMPPS and NPS, 2019). Probation practitioners are not usually expected to directly provide healthcare, as people on probation can access services in the community. However, practitioners should identify health-related drivers of offending and facilitate access to support for people on probation including via GP registration, identifying where community sentence treatment requirements (CSTRs) may be appropriate, partnership working on the Offender Personality Disorder Pathway, and by supporting continuity of care for people released from prison.

Whilst probation practitioners may record information about the health and social care needs of people on probation, needs are not always consistently identified and recorded. People on probation encounter many barriers to accessing health and social care, including as a result of dual diagnosis (a combination of mental illness and substance misuse needs) (Revolving Doors Agency, 2017; Sirdifield et al., 2019). Challenges resulting from high caseloads and shortages of resources make it difficult for probation practitioners to build trusting supervisory relationships, identify people’s health and social care needs and consider them in sentencing recommendations, and help people on probation to access the support that they need (Mullen et al., 2022).

One approach to measuring and improving health and the quality of health and social care provided is using quality indicators as part of a plan, do, study, act (PDSA) cycle (Mainz, 2003; Rushforth et al., 2015). These indicators are used in numerous settings, including within the criminal justice system (CJS), but no such measures are routinely used in probation practice in England and Wales. Consequently, there is a dearth of evidence to enable us to identify effective practice within probation practitioners’ health-related role, and to help the probation service to influence decisions around what health and social care is provided and how it is provided by sharing evidence of effective practice and areas where the quality of care could be improved for people on probation.

We are using a modified RAND method (Van Engen-Verheul et al., 2011) to develop quality indicators relating to the objectives in the probation health and social care strategy. Our aim is for the indicators to be suitable for routine use in probation practice to support health improvement amongst people on probation, and to measure and monitor the quality of care that they receive. This method has six stages: investigating what professionals working in the criminal justice and health field and individuals with lived experience of being on probation consider the characteristics of high-quality health and social care for people on probation to be, and how they think these characteristics could be measured (stages 1 and 2); a systematic scoping review to identify relevant existing standards and measures (3); combining findings from these stages to produce draft standards and quality indicators (4); professionals rating the draft indicators (5), and (6) professionals discussing the draft indicators and re-rating (amended) indicators following this. The highest scoring indicators are recommended for use in practice. In this paper, we present findings from the first two stages of this process.

## Methods

Ethics permission for the study was gained from the University of Lincoln (2021_6947) and the National Research Committee (2021-124).

A purposive sample of 15 professionals selected because of their roles in the criminal justice and health fields completed an online survey. The professionals had varying backgrounds including primary care, prison, public health, academic, probation, inspectorate and policy roles. Using open-ended questions, the survey aimed to ascertain perceptions of:

1. The characteristics of high-quality health and social care for people on probation – what characteristics (standards) should be measured?
2. How these characteristics should be measured – what quality indicators should be used?
3. If any quality indicators already exist within other parts of the CJS or in probation internationally that could be used within probation in England and Wales.

Following this, a focus group was held with a purposive sample of seven people to consider the same topics. Participants were selected as they had lived experience of being on probation and their contact with the CJS was driven by multiple unmet health and social care needs. The focus group was co-produced with people with lived experience, who worked with the researchers to design the topic guide and facilitated the discussion. Participants were asked about their experiences of discussing health and accessing care whilst on probation, what they thought probation practitioners should know about their health, what might support them to have honest conversations with probation about their health, and how probation could help them to meet unmet needs.

Data were analysed using thematic analysis (Braun and Clarke, 2006) by a team of academics and individuals with lived experience of the CJS. This approach was selected as it is flexible, allows inductive coding and is suitable for use in a team with varying levels of experience.

## Findings

We created eight themes from the professional panel survey to describe what participants considered the characteristics and measures of high-quality health and social care to be, and a ninth theme based on their feedback around what would facilitate adoption of the indicators.

We developed eight themes from the lived experience focus group to describe what participants thought the characteristics of high-quality health and social care provision for people on probation were and how these could be measured, and what was needed to facilitate trust and honest engagement with probation around health and social care needs.

There was considerable overlap between the themes created from the two sets of data, with joint agreement that person-centred approaches were essential. Consequently, the themes are presented together where appropriate below.

### Early identification of needs and need for service access

The professional panel data suggested that high-quality care is based upon an accurate understanding of individuals’ health and social care needs and options for addressing them. This would enable practitioners to tailor interactions with people on probation according to their individual needs, consider health needs in sentencing recommendations, and provide a basis for signposting people to community-based services. As detailed later, a trusting supervisory relationship was perceived to be key to enabling open discussion around unmet needs. The professionals felt it would be beneficial for probation to use consistent routine screening/assessments to identify needs including for mental and physical health issues, learning difficulties, neurodivergence and drug and alcohol issues:

‘Mental Health: early identification, assessment, treatment and support is exceptionally poor. Many individuals coming through the CJS present with underlying mental health and neurodiversity issues which in many cases are not taken into account or addressed leading to many being sent to custody when treatment and inpatient treatment would be the appropriate solution’ (Professional [P] 3).

‘Testing for general health in the population should be key too like blood pressure/diabetes and checking lungs if they are a smoker/drug taker etc. Often these patients neglect themselves so picking up disease processes early can help’ (P6).

‘In relation to neurodiversity, there is not a routine screening used to help identify people’s needs, this impacts on the ability of the probation service to deliver good quality services and meet the needs of people with autism and learning disabilities’ (P10).

Information about unmet needs could inform commissioning and resourcing decisions and thus potentially improve the accessibility of health and social care services for people on probation and contribute to reducing health inequalities. Currently however, there are structural barriers to this, as appropriate tools and data are not always available within case management systems, and even when assessments are completed using validated tools, the data are not always easily reportable (Richards, 2020).

Professionals suggested that indicators to measure this characteristic could involve recording completion of and findings from screening/assessments, including recording whether an individual needs to be registered with a GP, and whether someone is already accessing care or would benefit from being referred for support (including via CSTRs).

### Role for peer support/mentoring

Those with lived experience saw peer mentors as potentially having a role in identifying needs, arguing that people may be more comfortable discussing health with a peer mentor. Participants expressed frustration at having to repeat information that they had already shared at earlier stages of the criminal justice pathway (see ‘collaborative’ theme) and argued that if peers had a consistent relationship with someone throughout the pathway, they could advocate for that individual’s health needs and thus reduce the number of times they must repeat sensitive information. Peers may also be able to help an individual to build a positive relationship with a probation practitioner.

‘You’re trying to establish this relationship that isn’t quite possible in your position. So, allow like a peer mentor to have those conversations and relay them back to you and allow the peer mentor, it’s not that the peer mentor’s the probation officer, but let the probation officer do their job and let the peer mentor do theirs’ (Lived experience [LE] 4).

‘I think speaking to somebody that has been through it and knows how that person’s feeling will respond so much better to them than someone that’s there to tick a box and to just do their job’ (LE5).

### Collaborative working

The professional panel and those with lived experience discussed the potential for probation staff to use existing sources of information about health needs from partner services (‘collaborative’ and ‘information sources’ themes respectively). There should be clear information sharing agreements in place to improve people on probation’s access to support, and to ensure timely continuity of care for those being released from prison:

‘Robust information-exchange agreements and protocols between criminal justice partners – multi-agency ownership and responsibility in contributing to the assessment, planning, delivery and review of interventions to support positive mental health’ (P11).

Lived experience participants argued that having to repeat traumatic experiences can have a negative impact on the individual and cause them to ‘shut down’. As such, information that has been gathered by one service should be available to related services to reduce this issue:

‘How many professionals has that person gone through of getting to a point of sitting in a chair with a probation officer and you still don’t know what’s wrong? There’s been social workers, there’s been lawyers, there’s been solicitors, there’s been police, there’s been doctors, there’s been nurses, there’s been prison officers, there’s been the lot, how come you don’t know? Start communicating with each other, start integrating your services’ (LE4).

Another person with lived experience noted that people may not always recognise their health problem(s) and access to any previous professional assessments may therefore be useful rather than relying on self-report alone:

‘If somebody is actually ill, or in a position of mental health at that time, they might not realise that they are actually ill…I had a drink problem, I didn’t know it, I just carried on until something major happened’ (LE 3).

As well as inter-agency collaboration, high-quality care was also characterised by a collaborative approach to care planning between practitioners and people on probation:

‘Communication is key to this and involving people in their health and social needs planning so that it is fit for purpose and meeting their needs’ (P14).

Suggested measures of collaborative working were firstly monitoring whether care plans are shared between agencies, and the proportion of discharge summaries received by probation and/or GPs following an individual’s release from prison:

‘Prisons need to send good quality discharge summaries to the GP in a timely manner so the GP is aware what a patient is discharged on (regarding medications) and if the mental health team were involved and what referrals the prison team have made. Audit, audit, audit. Review how may discharge summaries were sent to GP for example’ (P6).

Secondly, creating a digital record or application containing information on health needs, services contacted or accessed and potential sources of support:

‘a digital version of these things and of the services and contact numbers so have a useful app that people can access and everything’s on there…you can see what they’ve done, you can ask them in like a note thing, how are you getting on is there anything else I can help you with?’ (LE4).

Thirdly, probation utilising the 28-day referral scheme within the RECONNECT programme that has been introduced to improve continuity of care for people released from prison in England and Wales.^2^

### Needs-led

The professional panel suggested that high-quality care was characterised by being tailored to individuals’ specific needs, that is based on health-needs data, to ensure that suitable services are available to address complex needs (such as dual diagnosis) at all levels of severity, rather than only individuals with a high severity of need being eligible to access them. Services should also offer flexible access routes:

‘The current indicators focus on those whose mental health issues cross the secondary care threshold. The majority don’t fit this level of care, but they remain highly vulnerable with multiple complexities. Could a standard be added to reflect this’ (P3).

‘Mainstream services can stigmatise people in the criminal justice system and treat them less favourably. So, an equitable access to services is important, with sufficient flexibility within acceptance criteria to engage well with people on probation and meet, holistically, the range of needs and co-morbidities that people present with. Intersectionality as well as complexity of needs should be considered and catered for too’ (P10).

Suggested measures of this were recording barriers to and/or experience of service access.

### Accessible

Many professionals highlighted the difficulties that people on probation currently face in accessing services such as waiting lists, strict eligibility criteria that leave people unable to access support, and a lack of flexibility to accommodate ‘chaotic lifestyles’. Existing difficulties were described as having been exacerbated by budget cuts and COVID-19.

‘Many individuals within this cohort are highly vulnerable but accessing services to support them is difficult. This is compounded if the individual is deemed not suitable to access secondary care services. Neurodiversity…Accessing statutory services to support these individuals is poor with long waiting lists to be seen’ (P3).

Collaborative working and informing commissioning through needs-assessments were seen as potential solutions. Additionally, the professionals said there should be clear, and evidenced routes for referral or self-referral to health and social care services from probation, which are simple to navigate (perhaps via a single point of contact) and offer timely access to support. Some barriers could be overcome by services being co-located within probation and available in locations which are convenient to people on probation:

‘Health services do not accommodate the needs of chaotic/unreliable offenders – discharging from services when appointments are missed. Services need to be more aware of the needs of offenders, be more flexible and recognise Probation Practitioners as equals/professionals with whom they can share information to ensure treatment/appointments are maintained…Good health care services (e.g. in mental health and substance misuse) are available at times and in places that offenders frequent – at/near probation offices or out in the community’ (P5).

Examples of current work to improve the accessibility of care were provided including mental health treatment requirements, liaison and diversion services, Health Trainers and bringing health provision into probation offices.

Suggested quality indicators for accessibility included having clear referral routes, and recording the number of CSTRs recommended at court, the numbers of referrals made and subsequent engagement with services, and the timeliness of access:

‘A standard which focused on the increased use of [Alcohol Treatment Requirements] ATR/[Drug Rehabilitation Requirements] DRR’ (P3).

‘Levels of referral and uptake of primary and secondary mental health services’ (P13).

‘We should be looking to deliver timely and proactive support, and prevention so that needs are identified early and can be addressed before they escalate leading to worse outcomes (and more costly intervention)’ (P4).

### (Positive) outcomes

The professional panel stated that attending health and social care services should produce positive outcomes, which should be recorded. We grouped the examples of desirable outcomes within the data into three categories. Firstly, simply that someone had gained access to services/support through appropriate routes:

‘Number of people on probation with a mental health care plan. Number of people on probation in receipt of social care support…Number of people on probation registered with a GP’ (P5).

‘Having indicators that can be audited to assess what is happening, e.g., how many patients are tested for Blood Borne Viruses/HIV etc and how many get treatment’ (P6).

‘Engagement with RECONNECT services to better plan for health and probation needs on release’ (P14).

This could potentially lead to a reduction in avoidable use of crisis care and Professional 1 suggested ‘*less presentation at A&E*’ as a potential outcome to measure here.

Secondly, people receiving a diagnosis and improved health and criminal justice-related outcomes:

‘Outcomes could include ability to live independently in work or education improved quality of life or not reoffending, less alcohol drug use, less medications…[less] suicides, less deaths. Quality indicators could be measurable practical outcome that is meaningful for the service users and helps them’ (P1).

‘Number of people who have a diagnosed mental health, substance misuse or neurological condition’ (P5).

Finally, the need to not only measure that a service had been accessed, but to also consider the quality of engagement with the service – for example, using measures of experience and satisfaction:

‘“Promote GP registration” is almost meaningless. A quality indicator of % registered with GP would be better but still does not capture whether the individual makes and attends appointments nor the quality of the interactions with the health professional’ (Professional 9)

‘Measures of satisfaction can be built in’ (P10)

Lived experience participants discussed the benefits of consulting people with lived experience in the development and measuring of outcomes, particularly in terms of seeking qualitative feedback on experiences of accessing support.

‘Things do need to be measured in terms of like having data and statistics…but also you need to be able to have…an independent…lived experience panel or board that help review, get that feedback, so you’ve got the human element as well as the statistical element’ (LE7).

### Evidence-based

The professional panel indicated that probation’s health-related practice should be evidence-based and linked to policy developments. An example of this was that the government strategy to combat drug-related crime should be better embedded into probation. Ideally, findings from the outcomes data described above would be used to continually improve probation and health and social care practice – ensuring that interventions producing positive engagement and change are promoted. As stated previously, service provision should be informed by needs assessment data. There should also be continuous learning from experience, for example in terms of suicide and self-harm – utilising information regarding near misses to help prevent future incidents:

‘And all interventions should be evidence informed so that we are confident that what we are doing will make a difference’ (P4)

‘Screening data should be systematically collected and aggregated to provide a more accurate assessment of the prevalence of neurodivergence to inform needs analysis and service planning at all levels of the CJS’ (P11)

No specific suggestions were made for how this characteristic could be measured.

### Foundations

#### Compassionate and person-centred approach

The data suggested that particular foundations needed to be in place for the above characteristics of high-quality health to be achieved. The first of these, described by both groups of participants was the need for a compassionate and person-centred approach in probation and health and social care practice (the ‘flexible, empathetic and person-centred approach’ theme from the professional panel data and the ‘probation service culture’ and ‘co-produced, person-centred and trauma-informed practice’ themes from the lived experience data).

Lived experience participants argued that if probation staff applied an empathetic approach to interactions with people on probation and focused on addressing the root causes of offending it would help individuals to feel listened to, supported and empowered. They argued that that this could not be achieved through minor changes, or ‘papering over cracks’:

‘I think the authorities look for something called a quick fix, it’s not possible…it’s been a messed-up circle for many, many years, the hole’s that deep it needs to start again from the core’ (LE3).

Collaborative working to co-produce goals was considered key to this – individuals should be able to explore their care planning options with staff. Independence and freedom of choice should be encouraged. Instead of focussing solely on risk of re-offending, there should be an equal focus on people’s strengths and improving their sense of self, helping them to work towards their goals and ambitions:

‘Really, it’s about providing the right environment and that comes down to asking the right questions of the individual in front of you. What they feel comfortable with, where they feel comfortable, they may even feel comfortable divulging that information through a text or through email and then being able to have the conversation face to face once the information is known’ (LE1).

‘Giving them that choice and just reaching out to let them know that you care and that you’re there, but letting that individual have that element of independence, which they have been stripped of for so long’ (LE4).

‘With probation officers and stuff having some sort of compassion in their approach towards what’s going on with service users in their lives, traumas that they’ve been through…helping them move forward’ (LE2).

Lived experience participants discussed an indicator in the form of a lived experience charter for probation:

‘A charter which awards probation offices a bronze, silver or gold award based on how they deal with health needs etc. based on a criteria created in a collaboration with lived experience and probation’ (LE1).

Participants described the fact that people on probation may struggle to attend appointments and may encounter stigma when accessing healthcare. There is a need to treat people on probation with respect and empathy, to take a person-centred approach to supporting individuals, and to understand the challenges and difficulties they may be experiencing because of their circumstances, or factors such as gender, ethnicity or past experiences. Support from probation and health and social care services should be flexible and take the individual’s personal circumstances into account including any trauma they may have faced and barriers to access such as low literacy levels:

‘Needs to be central and tailored to the client with an understanding of their back-ground and the challenges they will face once back in the community. Personality disorder needs tailoring for women and the whole support system understood’ (P1).

‘On average I believe services are poor quality because they do not address individuals holistically, and it can be very difficult to navigate systems to access support needed. Because of lower levels of literacy (and health literacy) in probation populations people often fall through the gaps’ (P4).

‘Some people might do better with conversations and then some literature in whatever way to take away, it’s just making sure it reaches everyone, is accessible to everybody’ (LE2).

Lived experience participants discussed changing culture through the training of new probation staff, and the potential role for lived experience in supporting such changes was another key theme (see ‘Appropriate Resourcing’).

#### Appropriate resourcing

Both groups of participants described the need for appropriate resources to be available within probation, and health and social care services to enable good practice – in terms of training, management and funding (including having sufficient personnel). Participants indicated that the breadth of probation training should be increased to ensure that staff have the knowledge to manage the range of health and social care issues that people on probation face within the boundaries of their role – that is probation staff are not expected to be experts in health, or to directly provide healthcare, but should be able to recognise health issues and signpost appropriately. Staff should be sufficiently trained in screening, suicide prevention, the specific needs of neurodiverse individuals, and trauma-informed care. A system should be in place to monitor and ensure that training takes place and is effective. Training should include lived experience input (also see ‘role for lived experience’):

‘We should be looking for increases in the: Number of probation staff appropriately trained in mental health, substance misuse, suicide prevention, neurodiversity support’ (P5).

‘The input of people with personal experience of neurodivergence into training was highly valued by those who had received it, and this should feature in any future training or awareness-raising programme’ (P11)

‘Well basically the training that everyone’s receiving now, trauma informed training. There’s so much more awareness of adverse childhood experiences, of multiple disadvantages of poverty, of mental health, of drug additions, so that’s what we need to be focusing our training on’ (LE1)

There is a need for effective leadership and for services to be well-funded and resourced. Recent budget cuts to health and social care services were problematic, and probation staff were perceived to be overloaded – something that peer mentors could potentially help to alleviate:

‘Management oversight was missing or poor in far too many cases – managers must address their own knowledge gaps and hold more robust conversations with practitioners, so there is a clearer understanding of effective and ineffective practice’ (P11)

‘OPD [offender personality disorder] pathway is funded and well established, demonstrating some positive outcomes following the interventions received by the dedicated OPD team’ (P3)

‘Substance misuse and probation services are not working well enough together. The cuts to commissioned service budgets (up to 40% in England) and the dissolution of co-located, multi-agency teams has worsened services for people on probation. Drug and alcohol needs feature for around half of the probation caseload, but staff training and pathway development are insufficient to underpin good service delivery’ (P10)

‘The feedback I’m getting back off the probation officers is we have enough to do, we are “we are not trained in this, we are not trained in that” so in some way I do feel sorry for them because they are scape goats sometimes, they’re being overloaded’ (LE3).

The scale of the structural challenges currently being faced by probation and health and social care services is clear from the literature (see e.g. Black, 2021; HMIP and CQC, 2021; Millings et al., 2023). We need to resolve complex workforce challenges and under-investment in services for high-quality care to be achieved.

#### Role for lived experience

Ensuring that people with lived experience are consulted, listened to, and included in the development of probation services was a high priority among the lived experience participants in relation to culture change and training as illustrated above. Participants also saw a lived experience role in front-line delivery of support as mentors and in developing and using quality indicators for health and social care. Potential measures of this characteristic included the proportion of training courses that included delivery from people with lived experience of probation and the development of a lived experience charter for probation:

‘You’ve got your own kind of mission statement and head office quotes, so it’s basically what are you doing to achieve those, and the lived experience charter could be something that I believe would 1. Strengthen the relationship between practitioners and people on probation, but I also believe it would be something that could change the culture of probation’ (LE1)

‘There must be at least a third if not more, people of lived experience on those panels to deliver the real trauma informed training that you need’ (LE4)

#### Building and maintaining a positive, trusting and consistent relationship

In our final underpinning characteristic/theme, lived experience participants suggested that for people on probation to feel comfortable discussing sensitive issues such as health with probation staff, they need to have a positive and consistent relationship with one probation practitioner where they establish sufficient trust and rapport to feel able to share sensitive information in their own time. Again, this is difficult to achieve given the structural challenges in the current climate. Participants shared their own experiences of frequently being passed between probation practitioners and how this reduced continuity of care and prevented them from developing a beneficial relationship:

‘You can’t ask direct stuff like that, you have to wait for them to come to you. You have to break the barriers down, they have to get to know you and as soon as they get to know and they think they can trust you, then it’s time you can help, until that time, and you’ve got a barrier up, nobody’s helping anyone’ (LE3)

‘Because the one thing that really is challenging when you’re trying to heal and you’re trying to establish these relationships of trust is the lack of consistency, you are constantly being handed to the next person to the next person to the next person, and… you don’t form a proper sort of bond, and yet you’re expected to sort of share out your whole entire, the most invasive and personal things about yourself. It’s like who are you? Why should I tell you?’ (LE4)

Having a positive relationship was also perceived to benefit the probation practitioner by helping them to better understand the needs of the individual:

‘On my last sentence with my probation, I had the same one [probation officer] from my pre-sentence report to the end of my license and that made a big difference because she got to know me, understand what my needs were and the rapport changed because, well it built basically and she was able to support me in some of the things that I needed…them getting to know you and understand you and taking that time to understand more of what’s going on with you more than just the offending, makes a difference’ (LE2)

Additionally, participants suggested that trust in this relationship could be enhanced through practitioners performing an advocacy role – not just signposting to services but helping individuals to access those services and following up afterwards:

‘Personally I’d like them to be with me every step of the way, you don’t just want them to think oh they’ve gone for that service, it makes me feel like they’ve just washed their hands of your issue, so you know…if it’s just asking about an appointment, how’s it going with that, is it helping’ (LE5)

### Facilitators for indicator acceptability

Finally, the professional panel data included ideas around how to facilitate the acceptability and effectiveness of the indicators that we intend to develop – indicators should be meaningful for people on probation, succinct, and simple to administer. They should also be measurable and enable useful comparison between groups. Furthermore, qualitative, as well as quantitative data, should be utilised so that processes required to produce good outcomes as well as the desired outcomes can be monitored and improved over time.

## Discussion

We are developing quality indicators to support health improvement amongst people on probation and to measure the quality of health and social care that they receive within the areas in the NPS health and social care strategy 2019–2022. Here, we present findings from our first steps towards this – exploring what professionals working in the criminal justice and health field and people with lived experience of being on probation think the characteristics of high-quality health and social care for people on probation are (what standards we should meet), and how these might be measured (what our quality indicators should be).

The value of quality improvement approaches that draw upon both professional and lived-experience perspectives to improve care quality has long been recognised in healthcare research and policy development work (see for example Backhouse and Ogunlayi, 2020; Siriwardena and Gillam, 2014). A strength of this research is that it is based on responses from professionals with heterogeneous backgrounds, and people with lived experience of being on probation. There was considerable overlap between the opinions of these two groups. The transferability of our findings is supported by how the themes created resonate with those in the wider literature.

We recognise that we are describing ideals which may be difficult to meet in practice due to structural barriers (which are well-evidenced as referenced in the ‘Findings’ section) such as staffing shortages within probation and healthcare settings, and a lack of central strategy, funding and evidence regarding how best to identify and meet the needs of people on probation. However, understanding the ideals is important – people on probation should be able to access care that meets their needs in the community, but currently provision falls short of that. Improvements are needed so that sufficient accessible and appropriate mainstream care is available to meet the needs of this population.

So, what did we learn about the characteristics of high-quality care and how these could be measured? The professionals working in the field and individuals with lived experience participating in the study agreed that ultimately, high-quality health and social care for people on probation should produce positive health and criminal justice outcomes. Therefore, our quality indicators should measure access to services and the outcomes of this e.g., changes in health status, reoffending, and individuals’ perception of their health. Ideally, reporting of indicators would include comparison between sub-groups to identify any differences in outcomes between groups based on individuals’ personal characteristics or types of health or social care need. Measures of the quality of engagement with services may also be beneficial as simply attending appointments may not be enough to produce a change. Moreover, it is important to recognise and record progress towards goals, for example using less drugs as a step towards becoming drug-free, or improved attendance at support services. Other important measures are individuals’ satisfaction with and experience of engaging with support.

To arrive at the point where health gains and positive criminal justice outcomes are achieved, several other key steps are needed. We need a culture within probation that promotes consistent supportive supervisory relationships and inter-agency collaboration. The data show the potential benefits of adopting a compassionate and person-centred approach within probation practice to facilitate more honest discussions of health and social care needs. Despite changing paradigms within probation practice as the service navigates shifts in the framing of its dual role of public protection and rehabilitation, the literature continues to remind us of the central importance of the supervisory relationship to achieving desistance (see for e.g. Dowden and Andrews, 2004; Rex, 1999). This relationship has been referred to as a ‘working alliance’. Studies have also examined the impact of this in relation to health outcomes for people on probation and on parole. Findings suggest that when the relationship is established early, and is consistent, supportive and characterised by trust, fairness and a collaborative approach to setting and achieving goals, this may lead to improved criminal justice and health outcomes (Bosker et al., 2020; Epperson et al., 2020; Mullen et al., 2022; Walters, 2016). Our findings suggest that the quality of this alliance could be enhanced by practitioners not only making referrals but acting as advocates for access and following up on the outcomes of engagement. These characteristics can be measured through indicators around the experience of service access and the supervisory relationship, introducing a lived experience charter, and measuring the number of supervisors that an individual has over a 12-month period.

Alongside this, the probation service requires sufficient resources to enable the consistent relationships described above and for training to enable practitioners to perform their health-related role well. Recent reports suggest that considerable work is needed in these areas as currently there are significant staff shortages and training needs to improve (Criminal Justice Joint Inspection, 2021; HM Inspectorate of Probation et al., 2021; HMIP and CQC, 2021; The London Assembly Police and Crime Committee, 2023). For example, an inquiry in London reported that ‘over-stretched probation staff did not have enough time to get to know individuals and thereby spot signs of mental health deterioration; and that diagnoses are not followed up with support’ (The London Assembly Police and Crime Committee, 2023: 29). There is a role for lived experience here in developing and providing training. Quality indicators should measure this contribution alongside the proportion of practitioners attending training.

In addition to collaborative working within a supervisory relationship, interagency collaboration is also needed – initially, to establish key structures and processes for inter-agency information exchange and to create clear referral routes from probation into health and social care services. A recent report emphasises the need for information exchange ‘with drug services in the community and in prison, and probation-led rehabilitative services in prison and in the community – in order to fully integrate and inform a resettlement and treatment plan for every person leaving prison’ (HMIP and CQC, 2021: 12). This is reflected within probation’s health and social care strategy. Quality indicators should measure the existence and use of these structures and processes.

The ability to exchange data on health needs supports another key characteristic of high-quality care – that it is needs-led. Commissioning decisions are based on local Joint Strategic Needs Assessments and at present, very few of these include data about the health and social care needs of people on probation (Revolving Doors Agency, 2017; Richards, 2020). Currently there are shortfalls in health and social care provision for the general population in the UK that have been exacerbated by budget cuts and COVID-19 (Black, 2021; HM Inspectorate of Probation et al., 2021; HMIP and CQC, 2021; Sirdfield et al., 2022). Added to this, and reflecting the wider literature, responses in our study highlight further obstacles that people on probation may encounter such as stigma, low literacy, location and opening hours for services and restrictive eligibility criteria. Ideally, health, social care and probation services should offer flexible support that is tailored to the individual. For example, flexible engagement routes (with due consideration of factors such as risks and confidentiality) and taking a trauma-informed approach (McCartan, 2020; Sirdfield et al., 2022). Whilst the probation service cannot directly control what services are available locally, potentially, the suitability and accessibility of services and continuity of care could be improved through routine collection and sharing of data between the probation service and commissioners. This has been recognised in numerous reports, including recent thematic inspections that recommend the use of a common definition of mental health across the CJS and a joint national memorandum of understanding to facilitate information exchange (HM Inspectorate of Probation et al., 2021). Needs data are also key to informing sentencing decisions including the potential use of CSTRs, as well as to inform resettlement planning including referrals from probation to health and social care services.

Therefore, it is essential that probation practitioners have access to appropriate screening and assessment tools alongside data from partner agencies to identify and record individuals’ health and social care needs and whether these are being met or whether an individual may benefit from a referral. Quality indicators should measure the existence and use of these tools together with what works well and any barriers to service access.

Participants also suggested that high-quality care is evidence-based and aligned with policy. Collecting and sharing data as part of a PDSA cycle would add to evidence base, enabling identification of good practice that could be spread, more efficient use of existing resources, and planning to address areas for improvement.

The voice of lived experience can contribute substantially to many of the above characteristics – in terms of an individual having a say in their own care planning, and roles for people with lived experience (such as peer mentor roles that previously existed in CRCs) in contributing to: training for probation staff, sharing information about individuals’ health needs as they progress through the CJS, delivering support, helping people to establish positive relationships with probation staff, and developing and using quality indicators.

In later stages of the wider study, we will conduct a systematic scoping review to identify existing quality indicators in use with adult criminal justice populations within the areas detailed in probation’s health and social care strategy that may be appropriate for use or easily adapted for use in probation settings and compare these to the characteristics of high-quality care identified here. We will then produce a list of potential quality indicators that will be rated by professionals in the field and then refined into a final set of indicators that could be routinely used in probation practice.

## Conclusion

The quality of health and social care for people on probation can be measured and improved through using quality indicators as part of a PDSA cycle. Data from professionals working in criminal justice and health, and people with lived experience of being on probation suggest two key foundations for high-quality health and social care for people on probation. Firstly, positive, trusting and consistent supervisory relationships facilitate open and honest conversations about health. Secondly, probation and health and social care practice is sufficiently resourced to meet people’s needs, and takes a compassionate and person-centred approach. High-quality care is based on an understanding of the probation population’s health and social care needs and tailored to meet individuals’ needs (offering flexible access routes and accounting for individual circumstances such as low-literacy levels, neurodiversity and past trauma). It also involves collaborative working between health, social care, and criminal justice agencies; is evidence-based and produces positive outcomes for attendees. The voice of lived experience is central to achieving high-quality care, including through people having a say in their own care planning, contributions to training, identifying needs, practice development (including developing and using quality indicators), and front-line delivery of support. We will conduct further research to develop quality indicators to measure these characteristics with a view to them being used routinely in the probation service.

## Notes

1 This was produced when probation was split into the National Probation Service and Community Rehabilitation Companies (CRCs) in England and Wales. Provision has since been unified into one service and a National Partnership Agreement for Health and Social Care for England has been developed since this study was undertaken.

2 See https://www.england.nhs.uk/commissioning/health-just/reconnect/ for more information on RECONNECT.
